# Cryptic *Streptococcus mutans* 5.6-kb plasmids encode a toxin–antitoxin system for plasmid stabilization

**DOI:** 10.3402/jom.v5i0.19729

**Published:** 2013-01-15

**Authors:** Anke Rheinberg, Izabela Jadwiga Swierzy, Tuan Dung Nguyen, Hans-Peter Horz, Georg Conrads

**Affiliations:** 1 Division of Oral Microbiology and Immunology, Department of Operative and Preventive Dentistry & Periodontology, RWTH Aachen University Hospital, Aachen, Germany; 2 Department of Medical Microbiology, RWTH Aachen University Hospital, Aachen, Germany

**Keywords:** *Streptococcus mutans*, plasmid addiction system, toxin–antitoxin cassette, RelBE, MazEF, HicBA, regulator of translation

## Abstract

**Background:**

In all *Streptococcus mutans* strains, 5–13% carry a 5.6-kb plasmid. Despite its frequency, little is known about its mediated functions with most of the information coming from a single study focussing on plasmid pUA140.

**Objective:**

Here, we describe the sequence and genetic organization of two *S. mutans* 5.6-kb plasmids, pDC09 and pNC101.

**Results:**

Based on PicoGreen dsDNA quantification and Real-Time quantitative PCR (RTQ-PCR), the plasmid copy number was found to range between 10 and 74, depending on the strain tested. In contrast to literature, we identified six instead of five open reading frames (ORFs). While the putative gene products of ORF1 (as a Rep-protein) and ORF2 (as a Mob-protein) could be confirmed as being identical to those from pUA140, the functions of ORF3 (unknown) and ORF 4 (possibly AtpE homologue) could not be further revealed. However, the product of ORF5 showed a fairly high identity (38–50%) and structural similarity (58–74%) to RelE of *Streptococcus pneumoniae*, *Streptococcus equi*, and *Streptococcus downei*. In addition, we identified a functionally corresponding ORF6 encoding a protein with 61–68% identity (81–86% similarity) to the *S. equi* and *S. downei* antitoxin of the RelB family. RelE and RelB together form a plasmid-encoded toxin-antitoxin (TA) system, RelBE_plas_. Despite its rather limited sequence similarity with chromosomal TA systems in *S. mutans* (RelBE_chro_, MazEF, HicBA), we found similar tertiary structures applying I-Tasser protein prediction analysis.

**Conclusion:**

Type II-toxins, as the plasmid-encoded RelE, are RNA endonucleases. Depending on their mRNA cleavage activity, they might 1) kill every plasmid-free progeny, thereby stabilizing plasmid transfer at the expense of the host and/or 2) help *S. mutans* enter a dormant state and survive unfavourable environmental conditions. Whilst a function in plasmid stabilization has been confirmed, a function in persistence under nutritional stress, tested here by inducing amino acid starvation, could not be demonstrated so far.

Plasmids present in many gram-positive cocci usually encode for a variety of biological activities, including resistance to antibiotics, heavy metals, production of and/or resistance to bacteriocins, metabolic properties, immunity, and factors of pathogenesis. However, a 5.6-kb (corresponding to 3.6 megadalton) plasmid with a G + C content between 31 and 34 mol% in *Streptococcus mutans* has unknown functions and is thus still designated as ‘cryptic’. Interestingly, in clinical populations, it shows a relatively constant prevalence ranging from 5 to 13% (1, 2). Almost all plasmid-containing strains of *S. mutans* produce distinct mutacins which are not directly plasmid-encoded (3, 4). Furthermore, *S. mutans* plasmids can be divided into two types classified by bacteriocin profile and restriction enzyme digest: Group I plasmids isolated from strains of individuals from African/Asian descent and Group II plasmids from Caucasians (5, 6). Efforts to transform plasmid-positive into plasmid-negative strains have all been unsuccessful (2, 4). This makes studies regarding biological functions difficult even though they give evidence of an inherent plasmid-stabilizing system. By sequence analysis, Zou et al. (7) previously found five open reading frames (ORFs) on the representative pUA140 Group I plasmid. Based on cloning experiments ‘stability determinants’ could be assigned to ORF 1 and 5. However, toxin–antitoxin loci, which are frequently present in plasmids to confer stability and maintenance, were not known at that time.

The primary aim of our study was to get a better insight into the frequency, heterogeneity, and function of *S. mutans* 5.6-kb plasmids. By testing 40 *S. mutans* strains applying a PCR with primers directed to conserved regions, we found four plasmid positive strains. The sequence analysis revealed a toxin-antitoxin (TA) system of the RelBE family with RelE (toxin) known for cleaving mRNA positioned at the ribosomal A site and drastically reducing translation (8). We further tested the hypothesis that plasmid-encoded TA systems (TA_plas_) in *S. mutans* could 1) compensate function of mutated and defect chromosomal TA modules (we further abbreviate TA_chro_) and/or 2) generate a plethora of TA genes facilitating dormancy and persistence of the host under (amino acid) starvation.

## Methods

### Bacterial strains, plasmids, and media

Forty strains of *S. mutans* were cultured under standard conditions on Columbia blood agar aerobically, with 10% CO_2_ at 37°C, and screened for the 5.6-kb (3.6 MDa) plasmid by PCR using the primer pair pF6/pR3 (Table 1). The four positive strains were DC09, DD09, NC101, and NC102 (all originated from the collection of D. Beighton, KCL Dental Institute, London, UK). A selection of plasmid-free strains, namely UA159, ATCC25175, NCTC11060, OMZ918 (Oral Microbiology, Zürich, Switzerland), KK21, R187 (both from the Biological Laboratory, University Hospital, Jena, Germany), 5DC8 (D. Beighton), and AC4446 (National Reference Centre for Streptococci, Aachen, Germany) were used for contrast.

**Table 1:** Amplification and sequencing primers for *S. mutans* 5.6-kb plasmids

Primer	Target	Sequence 5’→3’	Annealing-temperature (°C)
pFA	Amplification of region A	TTTAAGCGAACGACAAGGCT	57.0
pRA	Product 2,283 bp (position 5,512–2,158, including ds-origin)	TCTGCTTGTGTGCCACTTTC	56.5
pFB	Amplification of region B	TCATGGGCTTATCTGCGACG	59.6
pRB	Product 2,353 bp (position 1,879–4,232)	CATCCTTCATTTCGCCTCTT	55.0
pFC_hyper_	Amplification of region C (hypervariable)	AAGAGGCGAAATGAAGGATG	52.8
pRC_hyper_	Product 1,424 bp (position 4,213–5,637)	GAGGTTTTGGAGTGAGC	55.0
pF 1	Sequencing starting 293–314	CCCAAACGAACTACTAATAGCA	53.0
pF 2	754–771	ATAACAGACCGTGATATG	52.0
pF 3	2,299–3,318	ATTTTGCTCGTTCAACAGGG	56.7
pF 4	2,771–2,789	AAAAAGTACCCCAAATAGC	48.6
pF 5**	4,525–4,547	TGCGAATTATCAGCACTAAAACA	55.4
pF 6*	4,788–4,807	CATATATGGCACAAAATGGC	51.8
pR 1	5,512–5,531	AGCCTTGTCGTTCGCTTAAA	57.0
pR 2**	5,124–5,143	CATTTGGCAAGGTCACAGTC	55.4
pR 3*	4,964–4,986	TAAAGTAGATACACTAGTACACC	42.6
pR 4	3,748–3,767	TTGCCACCAATGTTTTGGGG	62.1
pR 5	1,674–1,695	TTGCTGATTTCCCTGAACCTGT	58.2

* Also used as screening PCR for plasmid presence in *S. mutans* strains.

** Also used for plasmid number determination using RTQ-PCR.

### Growth conditions for persistence experiments

The basal synthetic medium (BM) consisting of buffering salts, 0.8% glucose, 0.2% casaminoacid, 2 mM MgSO_4_, vitamins, and four essential amino acids (see below) were used to induce starvation. Amino acid starvation causes guanosine tetraphosphates (ppGpp and pppGpp, known as alarmones) and, secondarily, PolyP to accumulate (9). PolyP in turn is a signalling molecule that activates the Lon protease to digest ribosomal proteins and readjust and adapt translation rate to starving conditions (10). Lon also degrades antitoxin of the RelB-type so that the toxin (RelE), an endonuclease, clears ribosomes from mRNAs further supporting readjustment of translation rate (8).

Amino acid starvation was induced by two different strategies: 1) Naturally induced amino acid starvation: The amount of casaminoacid (from 0.2 to 0.02%) as well as the amount of essential amino acids (L-glutamic acid from 4 to 0.4 mM, L-arginine from 1 to 0.1 mM, L-cysteine from 1.3 to 0.1 mM, and L-tryptophan from 0.1 to 0.01 mM) was reduced. The BM-broths (10 ml) were inoculated with 100 µl of an overnight *S. mutans* (strains as above) preculture, incubated for 48 hours at 37°C in a 10% CO_2_ atmosphere, and then kept at room temperature (RT). On a daily basis, 10–100 µl samples were struck on Columbia agar to stop dormancy and test for viability. When strains missed growth on agar, the suspensions were concentrated in 1 ml by centrifugation and again samples were struck on agar. If the final culture failed to grow a single *S. mutans* colony, the strain was reported as ‘dead’. This way differences in starvation tolerance and persistence could be assessed for all strains.

2) Chemically induced amino acid starvation: *S. mutans* strains (UA159, ATCC25175, DC09, DD09) were grown in standard BM broth. At A_540_=0.2, serine hydroxamate (SHX), an analogue of serine, was added (0.5 mg/ml) for the induction of amino acid starvation by inhibiting the seryl-tRNA synthase. After further incubation for 4 hours at 37°C in a 10% CO_2_ atmosphere, the cultures were kept at RT for the rest of the experiment. Again daily, 10–100 µl samples were struck on Columbia agar to test for viability. The protocol was finished as described above.

### DNA preparation and sequence analyses

Plasmid DNA preparation, restriction enzyme digestion, and sequencing were performed by standard methods and as previously described (11). DNA sequencing was accomplished via an automatic sequence facility (Applied Biosystems 310 DNA sequencer, Applied Biosystems, Foster City, CA). The complete base composition of both strands of plasmids pDC09 and pNC101 was determined by sequencing and assembling PCR amplified stretches of the plasmid using a newly designed primer system (Table 1) and the Vector NTI advance 9.0 program (Informax, Invitrogen life science software, Darmstadt, Germany). For PCR amplification, the following conditions were used: 94°C for 2 min; followed by 35 cycles of 94°C for 45 s, annealing temperature (calculation based on Table 1) for 30 s, and 72°C for 45 s (amplicon <800 bp) or 90 s (amplicon >800 bp), respectively; with a final extension at 72°C for 5 min. DNA and protein sequences were analyzed with the software programs GeneDoc (www.psc.edu/biomed/genedoc), Pfam (Sanger Institute, Cambridge, England), MOTIFsearch (GenomeNet, Kyoto, Japan), Softberry (MolQuest, Mount Kisco, USA), EMBOSS (European Molecular Biology Open Software Suite, Cambridge, England), and I-Tasser (12, 13). Database searches were carried out through NCBI and EMBL with the BLAST and ORF Finder search programs.

### Determination of plasmid copy number

To determine the plasmid copy number of all four strains, pNC101 was selected for the preparation of standard reactions. Amplification products generated using the primer pFC_hyper_/pRC_hyper_ and pNC101 (Table 1) were purified using the Qiagen Purification Kit (Qiagen, Hilden, Germany) according to the manufacturer's instructions. Purified products were subsequently quantified with the PicoGreen dsDNA quantification kit (Quant-iT PicoGreen, Invitrogen, Darmstadt, Germany). Knowing the exact size of the amplicons and using the average molecular weight of a single DNA base pair, the measured DNA value could then be converted to target molecule numbers per microlitre. Dilution series of these PCR products were further used as calibration standards to measure DNA obtained from all *S. mutans* cultures with an unknown content of plasmids via Real-Time Quantitative PCR (RTQ-PCR) on a LightCycler 2.0 using the assay-primers pF5/pR2 (Table 1). For determination of total *S. mutans* cells, the glucosyltransferase-gene *gtf* was quantified by RTQ-PCR in parallel as previously described by Horz et al. (14). The calculated plasmid/*gtf*(B/C)–gene ratio per *S. mutans* strain then gave an estimate of the average number of plasmids per cell. Various dilutions were tested independently to increase the accuracy of the measurement (Table 2).

**Table 2:** Determination of plasmid number per cell in four *S. mutans* strains

Strain	Dilution of cells	*gtf*-copy numbers measured (two per cell, *gtf*B and *gtf*C)	Plasmid numbers measured	Resulting numbers of plasmid per cell	Average number of plasmids/cell
DC09	1:10	1.15×E6	1.56×E7	27	
	1:100	1.13×E5	1.40×E6	24	∼23
	1:1000	1.18×E4	9.02×E4	15	
	1:10000	1.15×E3	1.5×E4	27	
DD09	1:10	8×E5	1.42×E7	37,5	
	1:100	8×E4	2.21×E6	62,5	∼74
	1:1000	7.96×E3	3.87×E5	100,5	
	1:10000	8.14×E2	4×E4	100	
NC101	1:10	2.1×E6	1.71×E7	17	
	1:100	2.5×E5	1.61×E6	13	∼15
	1:1000	1.95×E4	1.5×E5	15	
	1:10000	2.1×E3	1.5×E4	14	
NC102	1:10	3.5×E5	2.05×E6	12	
	1:100	3.5×E4	1.67×E5	9,5	∼10
	1:1000	3.52×E3	5.78×E3	33	
	1:10000	3.61×E2	2×E3	12	

### Analysis of chromosomally encoded TA systems

All *S. mutans* strains possess at least two chromosomal TA systems, referred to as mazEF and hicBA, and additionally in some strains like UA159, DC09, NC101-relBE (15, 16). We designed the following primers to amplify chromosomal TA cassettes in plasmid positive and negative strains for sequence and mutation analysis: mazEF-F 5’ AATAGACACAGAAATAGGAGGT 3’, mazEF-R 5’ GATTTCAGTAGAAGTTTAACTG 3’; relBE_chro_-F 5’ GGTATAATAGATATAAAAGGAGTT 3’, relBE_chro_-R 5’ AACAGGAAGAACTACCTTTGAG 3’; Hic-F 5’ CGCTAGAAATCAATCTTTCTA 3’, Hic-R 5’ TAGTTTCTAAATCAGGGGCT 3’. For PCR amplification, the following conditions were used: 94°C for 2 min; followed by 32 cycles of 94°C for 30 s, 48°C for 30 s, and 72°C for 45 s; with a final extension of 72°C for 5 min. DNA and protein sequences were analyzed using the software programs mentioned above and the TADB as well as the GenBank database.

## Results

A total of 40 different *S. mutans* strains were initially tested for the presence of a plasmid by PCR-amplification of a conserved 198 bp fragment using primer pair pF6/pR3 (Table 1). Four strains (10%) tested positive (confirmed by sequence analysis of the PCR-products) of which DC09 and NC101 were selected for an entire sequence analysis of their plasmids pDC09 and pNC101 and the resulting sequences deposed in GenBank under accession numbers HQ156229 and HQ156230. However, all four plasmid carriers were subjected to plasmid copy number determination.

### Physical map of plasmids pDC09 and pNC101

**Fig. 1:**
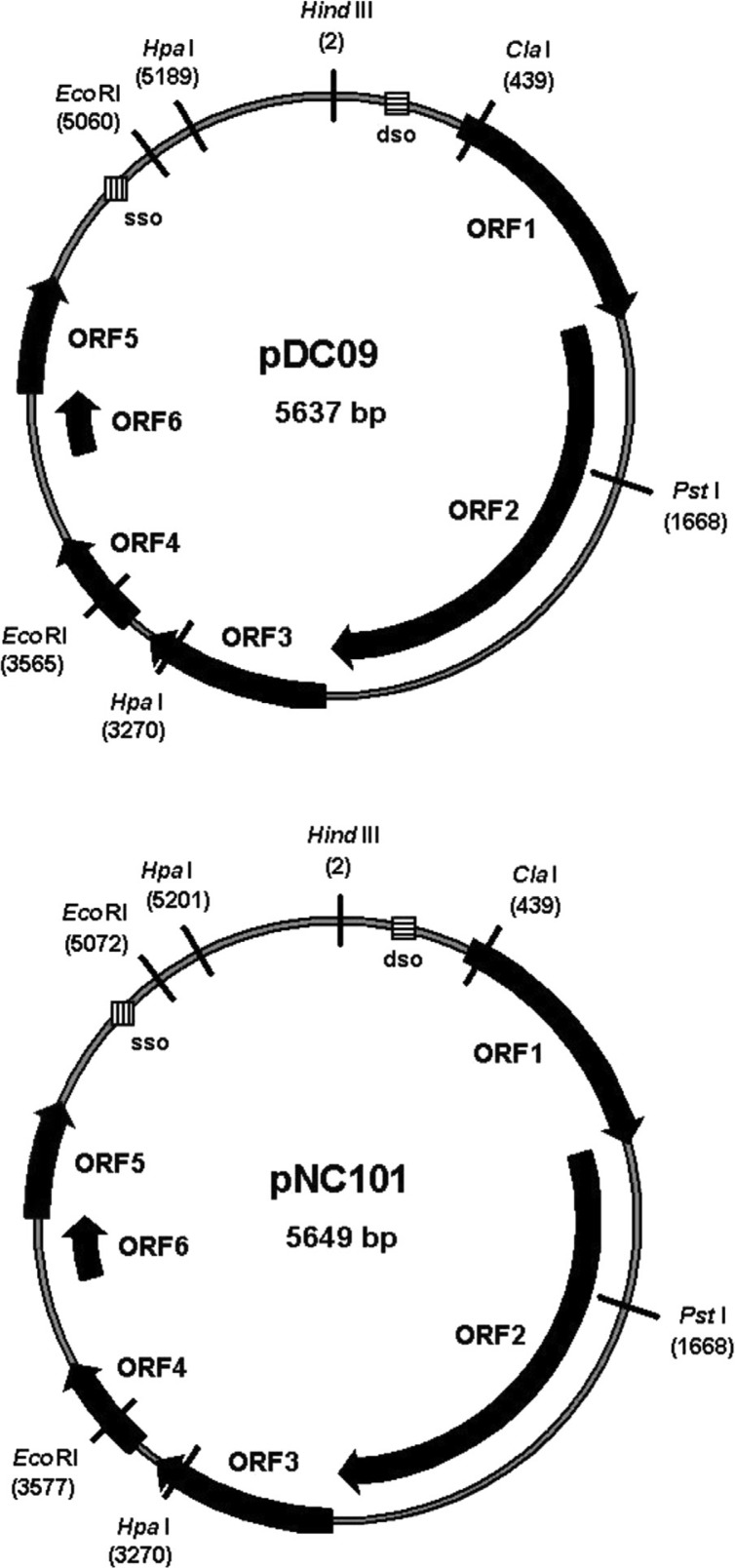
Physical map and organization of plasmids pDC09 and pNC101.

Both plasmids, pDC09 and pNC101, were found to contain identical restriction sites to the reference plasmid pUA140. They exhibited both single (e.g. *Hin*dIIII [position 2], *Cla*I [439], *Pst*I [1668], *Nde*I [2,247], *Afl*lI [3,082], *Xba*I [4,621], *Esp*I [4,473], and *Xcm*I [3,750]) as well as multiple restriction sites (e.g. *Hpa*I [3,270; 5,189], *Afl*lII [2,542; 5,415], and *Eco*RI [3,565; 5,060]) (Fig. 1 with selected restriction sites). With the exception of *Hind*III and *Eco*RI, which were tested experimentally by restriction analysis, the other sites were deduced from *in silico* analysis. The position of each restriction site is given according to pDC09 positions (in brackets). The exact size of pDC09 was 5,637 bp and 5,649 bp for pNC101, which is consistent with earlier estimations of related *S. mutans* plasmids (1, 2, 17, 18) and especially consistent with the size of pUA140 in the study of Zou et al. (7). The G + C content of pDC09 and pNC101 was not homogenous over the entire plasmid, approximately 32.6% (67.4% A + T) and 32.9% (67.1% A + T), respectively.

We found six ORFs encoding peptides larger than 90 amino acids (aa). All ORFs are located on the same strand with the following lengths of corresponding peptides: ORF1 (258 aa), ORF2 (555 aa), ORF3 (188 aa), ORF4 (105 aa), ORF5 (120 aa), and ORF6 (92 aa).

### Coding regions

#### ORF1

*In silico* analysis revealed that ORF1 in both plasmids was located between position 397 and 1,170 (according to the pUA140 reference sequence numbering (7)), thus 774 bp in length and encoding for a 258 aa Rep-protein. A Softberry search for promoter sequences revealed the -35-Box (5′TTCACA3′), a -10-Box (5′TAGTTATAAC3′), and a Shine-Dalgarno (SD) sequence starting with position -4 (5'AGGA3’) upstream from the ORF1 start codon ATG. Analysis of the amino acid sequence deduced from ORF1 of pDC09 and pNC101 gave results identical to the corresponding pUA140 sequence and showed similarity with the *Streptococcus thermophilus* Rep-protein. It demonstrated two motifs (II and III) universally associated with Rep proteins involved in rolling circle replication (RCR). Motif II may be involved in the binding of essential cofactor metal ions, such as Mg^2 +^ and Mn^2 +^. Motif III is thought to be involved in DNA binding.

#### ORF2

The ORF2 in both plasmids was located between position 1,158 and 2,822, thus 1,665 bp in length, and encoding for a 555 aa Mob-protein. The search for promoter sequences revealed the -35-Box (5’ TTTCAA 3’), a -10-Box (5’ ATATAGAAT 3’) and a potential SD-sequence starting with position -14 (5'AGGA3’) upstream from ATG. The deduced amino acid Mob-sequence (at its N-terminus) was clearly related to TraD proteins and to the c109099 superfamily represented by P-loop-NTPases. The TraD protein performs an essential coupling function in conjugative secretion systems. This protein connects the inner membrane of the cell–cell contact site with the relaxosome-plasmid DNA complex. Members of this superfamily are characterized by conserved nucleotide phosphate-binding motifs, also referred to as the Walker A motif and the Walker B motif.

The 3’ end of ORF1 and the 5’ start of ORF2 were found to share 16 nucleotides (5’ ATGGACAACAATTTAG 3’). Within ORF1, a 38 nucleotide sequence (5’ TTATATCTCTTGGATAAATTTAGGGTATTAGATGCTAA 3’) was found to be an almost inverted repeat structure of an ORF2-motif (5’ TTAGCAACTGTTACACTAAAATTAGCTAAAAAATATAA 3’). Its function could not be revealed but may be associated with the DSO repeats (see below).

#### ORF3

The ORF3 in both plasmids was located between position 2,838 and 3,401 (564 bp in length) and encoding for a 188 aa protein with an unknown function. The Softberry search revealed the -35-Box (5’ TAGTAA 3’), a -10-Box (5’ TTTTAAAAT 3’), and a potential SD-sequence starting with position -8 (5'AGGA3’) upstream ATG. However, even extensive search for homology in GenBank and other databases could not reveal any potential function of protein 3 or homology except the pUA140 homologue.

#### ORF4

The ORF4 was located between position 3,465 and 3,779 in pDC09, and between position 3,477 and 3,791 in pNC101, because of a 12 bp insert in the promoter region. Both fragments are 315 bp in length and encode for a 105 aa protein. The search for promoter sequences revealed the -35-Box (5'TATCTA 3’), a -10-Box (5’ TGATATCAT 3’), and a potential SD-sequence starting with position -6 (5'AGGA3’) upstream from the ORF4 start. In accordance with Zou et al. (7), we found that the putative protein 4 had a certain degree of similarity to the putative H^+^ transporting ATPase subunit E (*atp*E) in *Archaeoglobus fulgidus* (GenBank Accession No. B69395, 23% identity, 57% similarity). Since the proton translocation complex confers tolerance to low pH aciduricity (19), a plasmid-borne version of one of the ATPases may complement or replace the intrinsic protein thus altering or even enhancing ATPase function.

#### ORF5

The ORF5 was located between position 4,230 and 4,589 in pDC09 and between position 4,242 and 4,601 in pNC101, respectively in both cases, 360 bp in length and encoding for a 120 aa protein. No promoter sequences were revealed. Search for homology at the protein level revealed protein 5 to be involved in plasmid stabilization (super family cl11422). It shows a high identity (38–50%) and similarity (58–74%) to proteins of *Streptococcus pneumoniae* (hypothetical protein, GenBank No. ZP_01832116.1), *Streptococcus equi* (plasmid stabilization system, No. YP_002747188), and *Streptococcus downei* (addiction module toxin No. ZP_07726556.1, plasmid stabilization system No. ZP_07725301.1). This protein is involved in stabilization of plasmid transfer as it functions as a stable toxin of the RelE family.

#### ORF6

Finally, ORF6 was located between position 3,977 and 4,264 in pDC09 and between position 3,989 and 4,273 in pNC101, respectively, in both cases, 288 bp in length and encoding for a 92 aa protein. The search for promoter sequences revealed the -35-Box (5’ TTGCAC 3’), a -10-Box (5’ TTATATAAT 3’), and a potential SD-sequence starting with position -22 (5'AGGA3’) upstream from the start. Search for homology at the protein level revealed (putative) protein 6 to belong to the RelB superfamily cl01171 which was also involved in plasmid stabilization, with 68% identity to the corresponding protein of *S. equi* 4047 (GenBank No. YP_002747189) and 61% identity to *S. downei* F0415 (GenBank No. ZP_07725256), respectively.

### Rolling circle replication of pDC09 and pNC101

Rolling circle replication (RCR) plasmids typically share a number of common features. This includes a double- and single-stranded origin (DSO and SSO), respectively, a gene encoding for a replication (Rep) protein, and a single-stranded intermediate. Furthermore, the *nic* region is highly conserved among replicons of the same family while the *bind* loci usually vary (20, 21). The sequence 5’-CAAACGAACTACTAATAGCAGTAA-3’ on the coding strand from 296 to 319 bp of both plasmids pDC09 and pNC101 was identical or at least remarkably similar to the *nic* region of pUA140 (7), pT181 (22), and that of other plasmids or phages from the same plasmid family (20, 21). In particular, the nucleotide sequence 5’ CT/AATAGC 3’ between bases 307 and 314, which includes the *nic* site (T/A), was identical to the consensus sequence within the origins of pT181-like replicons. The putative DSO region contains several inverted repeats capable of generating hairpin structures, including one large hairpin with the *nic* site downstream. As the DSO usually lies upstream of or within Rep (23, 24) and the location of the *nic* site (T/A) in pDC09 and pNC101 was 89 bp upstream of the ORF1 start codon, further evidence exists that ORF1 encodes for the Rep protein.

Analysis of DNA sequence structure revealed a region between 4,998 and 5,174 on the encoding strand of pDC09 (5,011 and 5,192 on the encoding strand of pNC101, respectively), with a high level of secondary structure upstream of the *Rep* gene and close to the high G + C region. This agrees with the fact that the SSO region, although less conserved than that of the DSO region, usually resides within a region of strong secondary structure located upstream of the coding sequences for the *Rep* gene. Within this region, the hexanucleotide sequence 5’-TAGCGT-3’ was located as part of the stem of a large hairpin structure presumably acting as the site of second-strand initiation (ssi) and transcriptional terminator for the synthesis of an RNA primer (23).

### Determination of plasmid copy numbers

Purified and standardized plasmid PCR products were quantified with the PicoGreen dsDNA quantification kit as described above using different dilutions. A *gtf* B- and *gtf*C-targeting RTQ-PCR was applied to assess the number of *S. mutans* cells. The results are given in Table 2 calculated from different dilutions of pDC09, pDD09, pNC101, and pNC102. Whereas strains NC101 and NC102 exhibited medium copy numbers of 10 and 15, the copy numbers of strains DC09 and DD09 were 23 and 74. The latter might be very high which could be due to a high multiplication rate of host cells at the time tested. Regardless, the associated number of TA systems significantly increases the RNA endonuclease potential of the plasmid carriers.

### Sequence analysis of chromosomal TA systems in plasmid positive and negative strains

**Fig. 2:**
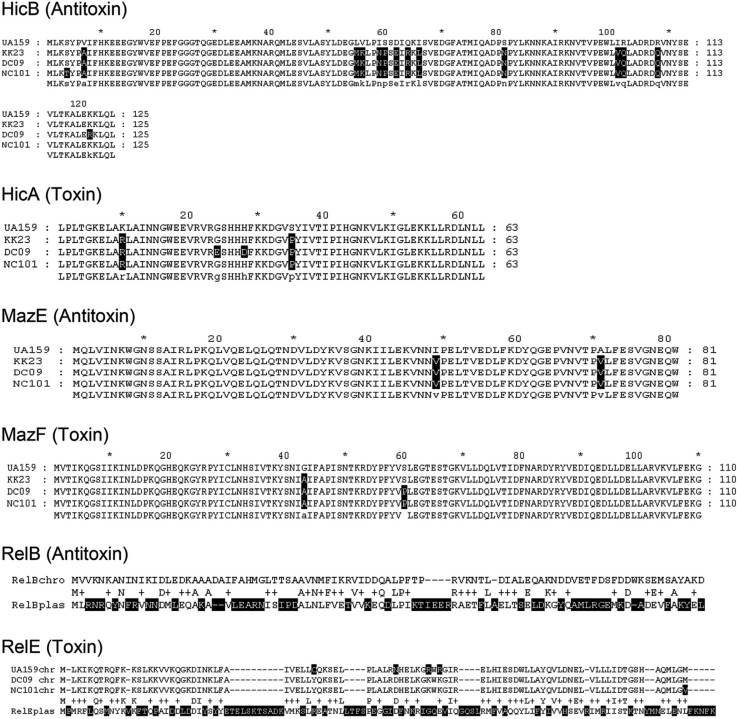
Amino acid sequence comparison of three chromosomally encoded TA systems (HicBA, MazEF, RelBE) between plasmid carrier strains DC09/NC101 and plasmid-free strains UA159/KK23. None of the alterations are typical for plasmid carriers or non-carriers or were found to be essential for the principal toxin or antitoxin function. In case of RelB, the plasmid-encoded version (RelB_plas_ of DC09 and NC101) has little similarity with RelB_chro_ (of UA159, DC09, NC101, all identical). In case of RelE, the plasmid-encoded version (RelE_plas_ of DC09 and NC101) has little similarity with the RelE_chro_ of UA159, DC09, and NC101. However, we found similar tertiary structures applying I–Tasser protein prediction analysis (Fig. 3).

**Fig. 3:**
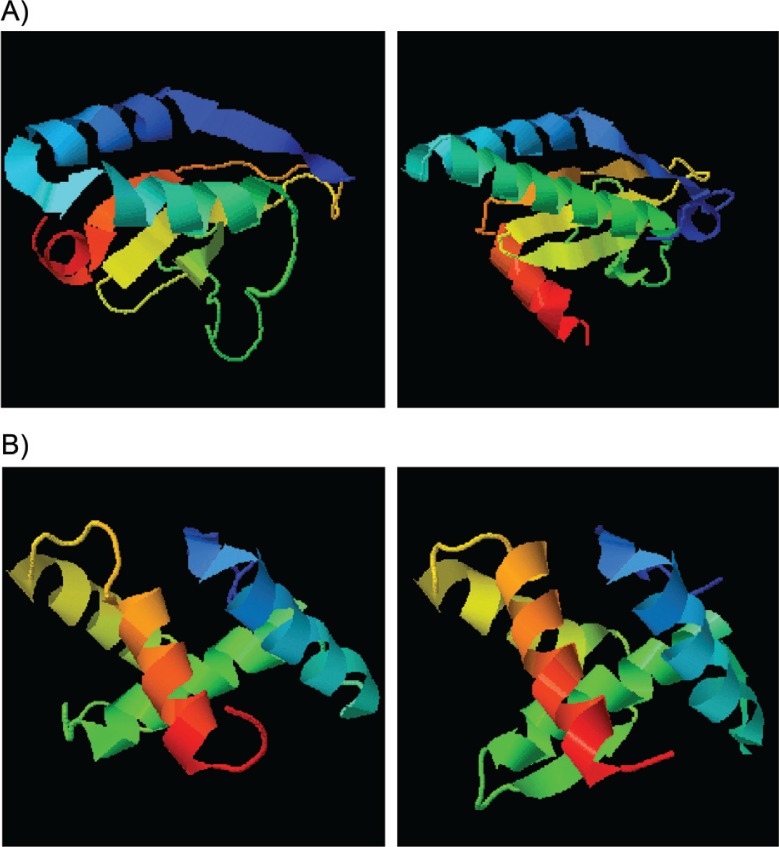
Protein prediction model based on I-Tasser, A) left: RelE_chro_ UA159, right: RelE_plas_ DC09/NC101; B) left: RelB_chro_ UA159, right: RelB_plas_ DC09/NC101. Source: http://zhanglab.ccmb.med.umich.edu/I-TASSER/ (12, 13)

As shown in Fig. 2, a number of differences in the chromosomal TA systems (hicBA, mazEF, and relBE_chro_) were found between strains. However, none of the resulting differences in amino acid sequence was found exclusively in plasmid carriers (DC09 & NC101) or non-carriers (KK23, UA159) and were essential for toxin or antitoxin function. Unexpectedly, the sequence homology between relE_plas_ and relE_chro_ and also between relB_plas_ and relB_chro_ was relatively low. However, as mentioned by Guglielmini and van Melderen (25), it is known that some toxin-superfamilies exhibit dramatic sequence diversities but have a similar tertiary structure. By running the I–Tasser protein prediction analysis, we were able to confirm this assumption for both RelE and RelB (Fig. 3).

### Survival under amino acids starvation

After incubation of *S. mutans* strains in BM-medium with 1) reduced casaminoacid and essential amino acids concentrations (i.e. 10% of standard concentration) or 2) with SHX as a starvation stress inductor, the cultures were monitored for viability daily.

Cleary, neither the medium copy number strains NC101 and NC102 (with 10–14 days) nor the high copy number strains DC09 and DD09 (with 10–14 days) persisted significantly longer than the plasmid-free *S. mutans* representatives (7–24 days).

## Discussion

We analyzed the genetic architecture of two new *S. mutans* 5.6-kb plasmids, pDC09 and pNC101. Both were highly similar to pUA140 and fall into what Zou et al. designated as the ‘Group I plasmids’ (7). They are usually found in *S. mutans* strains of humans of African or Asian descent. DC09 and NC101 were isolated in London making such an origin – even if not documented – plausible. With this evolutionary significance together with the inherent stability, these plasmids might be a good marker for *S. mutans* (and for its human host) evolutionary history.

Complementing the pUA140 data of Zou et al. (7), we found six ORFs which are located on the same DNA strand and encoded six proteins with a length between 92 (ORF6, product = antitoxin) and 555 (ORF2, product = Mob protein) amino acids. No meaningful sequence homologue was found for ORF3. As shown for pUA140, the plasmids replicate by RCR and belong to the pT181 family based on similarities in the Rep protein sequence (ORF1), the DSO, and the secondary structure-rich SSO (7). It is also noteworthy that DSO and SSO flank a high G + C region with many secondary structures, which supports replication initiation and termination. The Mob protein encoded by ORF2 plays a role in plasmid stability.

RelE (encoded by ORF5) and RelB (encoded by ORF6) form a toxin–antitoxin system (type II loci). In addition, the terms ‘poison/antidote system’ and ‘plasmid addiction system’ are used in literature. The corresponding genes, *relE* and *relB*, are usually under the control of the same promoter that we found located upstream of *relB*. RelE, an RNA endonuclease, is stable and terminates global translation through digestion of mRNA positioned at the ribosomal A site. RelB, as a counterpart, is unstable. It forms complexes with RelE, blocking the endonuclease activity and repressing its own promoter. Progenies that inherit a plasmid copy at cell division grow normally. In contrast, cells that do not inherit a plasmid copy still carry the stable toxin in the cytoplasm while the antitoxin is degraded. This leads to the selective post-segregational killing (PSK) of the plasmid-free progenies, but a dormant state of these cells should also be considered. In this way, TA loci prevent the proliferation of plasmid-free cells in growing bacterial cultures, leading to what is called ‘plasmid addiction’. In addition and according to the ‘selfish theory’, TA systems allow a conjugative plasmid to outcompete a second and TA-negative plasmid belonging to the same incompatibility group (26). Mathematical models demonstrate that the PSK phenomenon allows the propagation of TA systems in bacterial populations, independent of their original frequencies. This might provide a rational explanation for the evolutionary success of TA systems (27) and lead to the assumption that TA + plasmids might have an advantage over TA-plasmids under certain conditions (28–30).

A question yet to answer is if there are any links or interactions (cross reactivity, dosage effects) between TA_plas_ and TA_chro_, the latter involved in persistence under unfavourable conditions. Leung and Lévesque demonstrated that ectopic expression of RelBE_chro_ or of MazEF caused cell growth arrest and forms persisters under stress, in their case antibiotic treatment (31). From other studies, it is known that under amino acid starvation RelBE_chro_ reduces the global level of translation to save amino acids for the most essential housekeeping processes. As this is a mechanism present across bacterial phyla, cross reactivity must be considered as feasible. In any case, RelE is a global regulator of translation and is activated by stress via alarmones and via Lon proteases (digest of antitoxin), finally inducing mRNA cleavage (8, 9, 32). According to Gotfredsen and Gerdes (33), the chromosomal *relBE* locus stabilizes plasmids as efficiently as the plasmid-borne *relBE* loci confirming a kind of trans-activity. Recently, the same group showed that successive deletion of 10 different TA loci in *E. coli* progressively reduced the level of persisters, demonstrating a dosage effect (8).

We also tested the hypothesis that RelBE_plas_ compensates a dysfunction of corresponding TA_chro_ modules and is therefore beneficial or even indispensable for certain *S. mutans* strains. As shown by Lemos et al. (32), *S. mutans* UA159 has at least two TA loci on the chromosome (*relBE* and *mazEF*) plus a *hicBA* module as Song et al. recently found (16). In our study, we compared the amino acid sequences corresponding to all three loci between plasmid carrier (DC09, NC101) and non-carrier strains (UA159, KK23). Some alterations were found but none of them seem to be unique for plasmid carriers or non-carriers or accumulating in consensus regions essential for toxin or antitoxin function (Fig. 2). Altogether, in the case of RelB and RelE, the plasmid-encoded proteins show limited homology to their chromosomal pendants in their primary structure. However, when comparing the tertiary structures (Fig. 3), similarities become obvious and a grouping into the same family seems justified. Based on the three-dimensional structure, trans-activity of Antitoxin_plas_ inhibiting Toxin_chro_ and of Antitoxin_chro_ inhibiting Toxin_plas_ is conceivable.

The present study provides complete sequence information of two additional 5.6-kb *S. mutans* plasmids and revises the function of ORFs. It provides evidence that the ORF5 product belongs to the RelE (toxin) and that the ORF6 product belongs to the RelB (antitoxin) family. After comparing chromosomal and plasmid TA modules and corresponding protein structures, we speculate, depending on their netted endonuclease activity, whether they might 1) kill every plasmid-free progeny and stabilize plasmid transfer at the expense of the host and/or 2) help *S. mutans* to enter a dormant state and survive unfavourable conditions, as is known for chromosomal systems of the same kind. A function in plasmid stabilization has been confirmed: the 5.6-kb plasmids appear to be extremely stable in their *S. mutans* host as indicated by the apparent difficulty in curing strains of the plasmid according to the literature (3, 18, 34, 35) and our observations. The stability of such plasmids makes them attractive as cloning/expression vectors.

A function in persistence, tested here by inducing amino acid starvation using two methods, could not be confirmed. Clearly, further experiments with modified environmental factors (pH, temperature, oxygen, substrates, etc.) are needed to clearly exclude or confirm any advantage in the persistence of plasmid-carriers.
